# A case report: Pathological complete response to neoadjuvant lorlatinib for Epithelioid inflammatory myofibroblastic sarcoma with EML4-ALK rearrangement

**DOI:** 10.3389/fphar.2024.1401428

**Published:** 2024-07-31

**Authors:** Yang Zheng, Fanfei Zhao, Yaqian Ren, Yaran Xue, Bing Yan, Chun Huang

**Affiliations:** ^1^ Department of Thoracic Medical Oncology, Tianjin Medical University Cancer Institute and Hospital, National Clinical Research Center for Cancer, Tianjin, China; ^2^ Key Laboratory of Cancer Prevention and Therapy, Tianjin’s Clinical Research Center for Cancer, Tianjin, China; ^3^ Department of Oncology, Tianjin Fourth Central Hospital, Tianjin, China; ^4^ Department of Precision Oncology, Tianjin Cancer Hospital Airport Hospital, Tianjin, China

**Keywords:** EML4-ALK, Epithelioid inflammatory myofibroblastic sarcoma, neoadjuvant treatment, lorlatinib, pathological complete response

## Abstract

Inflammatory myofibroblastic tumor (IMT) is a rare tumor originating from mesenchymal tissue. Epithelioid inflammatory myofibroblastic sarcoma (EIMS) represents a rare and particularly aggressive variant, associated with a worse prognosis. Almost all EIMS cases exhibits activating anaplastic lymphoma kinase (ALK) gene rearrangements, which suggests that EIMS patients may potentially benefit from treatment with ALK tyrosine kinase inhibitors (TKIs). We presented a case involving a 34-year-old woman who was diagnosed with mediastinal EIMS and had a rare echinoderm microtubule-associated protein-like 4 (EML4) -ALK fusion. Following 15 months of neoadjuvant lorlatinib treatment, the patient underwent a complete surgical resection, resulting in a pathological complete response. Given the heightened risk of postoperative recurrence associated with EIMS, the patient’s treatment plan included ongoing adjuvant therapy with lorlatinib. As of the present moment, the patient has achieved an overall survival of over 2 years with no observed tumor recurrence. Consequently, the case offers valuable clinical evidence supporting the potential benefits of neoadjuvant lorlatinib treatment for ALK-positive locally mediastinal EIMS patients, with a demonstrated tolerable safety profile.

## 1 Introduction

Inflammatory myofibroblastic tumor (IMT) is a rare mesenchymal tumor with intermediate malignant potential. It comprises myofibroblastic spindle cells along with an inflammatory infiltrate (Coffin et al., 2007; Sukov et al., 2007). Epithelioid inflammatory myofibroblastic sarcoma (EIMS) represents a highly aggressive variant of IMT, characterized by the presence of epithelioid-to-round cells (Marino-Enriquez et al., 2011). Complete tumor excision is considered the preferred treatment option (Marino-Enriquez et al., 2011). Traditional radiotherapy and chemotherapy have shown limited benefits in the treatment of EIMS (Fang et al., 2018; Cheng et al., 2024). There is potential for benefit from targeted therapy in EIMS cases, particularly because these tumors often involve anaplastic lymphoma kinase (ALK) fusion (Kurihara-Hosokawa et al., 2014; Lee et al., 2017). Targeted therapies can specifically target the genetic or molecular abnormalities driving the cancer, which may lead to better outcomes for patients with EIMS.

ALK fusion was initially described as an oncogenic driver in anaplastic large cell lymphomas by Morris and colleagues (Morris et al., 1994). Targeted therapy against ALK alterations use tyrosine kinase inhibitors (TKIs) to treat ALK-positive tumors, most notably in non-small cell lung cancer (NSCLC) (Fukui et al., 2022). The most common fusion partners for ALK in EIMS cases are ran-binding protein 2 (RANBP2) and ribosome binding protein 1 (RRBP1) (Li et al., 2023). Lorlatinib, as a third-generation ALK TKI, has shown substantial activity. Notably, it has demonstrated superior efficacy compared to first-generation and second-generation ALK TKIs in the treatment of NSCLC (Solomon et al., 2018). Here, we present a case where lorlatinib was used as neoadjuvant treatment for EIMS.

## 2 Case description

**FIGURE 1:**
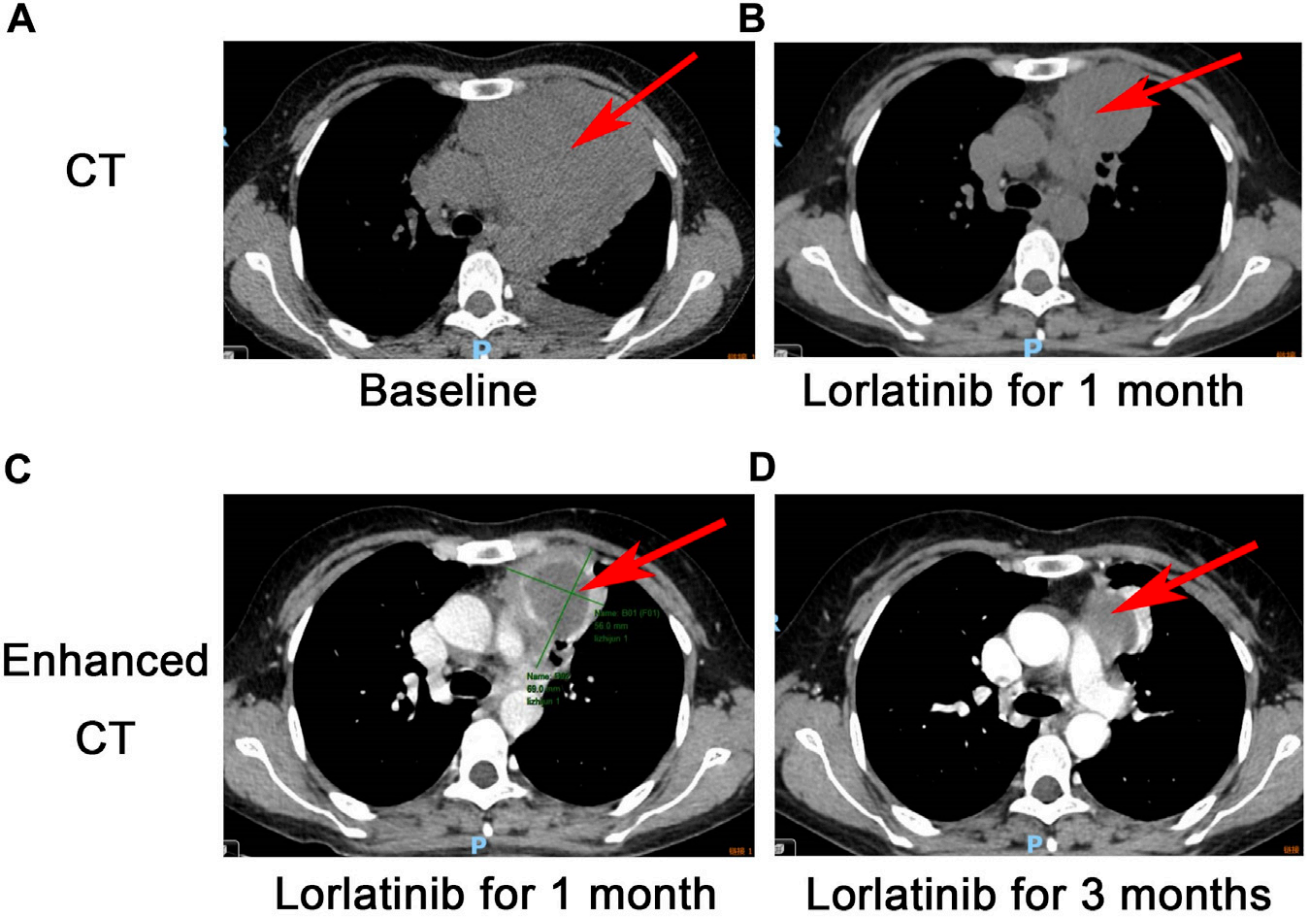
CT scan for EIMS: A large mass with unclear boundaries in the left anterior mediastinum measures 10.1 * 9.4 cm **(A)**, a partial response was revealed after 1 month following lorlatinib **(B)**, Comparison of contrast-enhanced CT of the chest at 1 month **(C)** and 3 months **(D)** after treatment with Lorlatinib.

**FIGURE 2:**
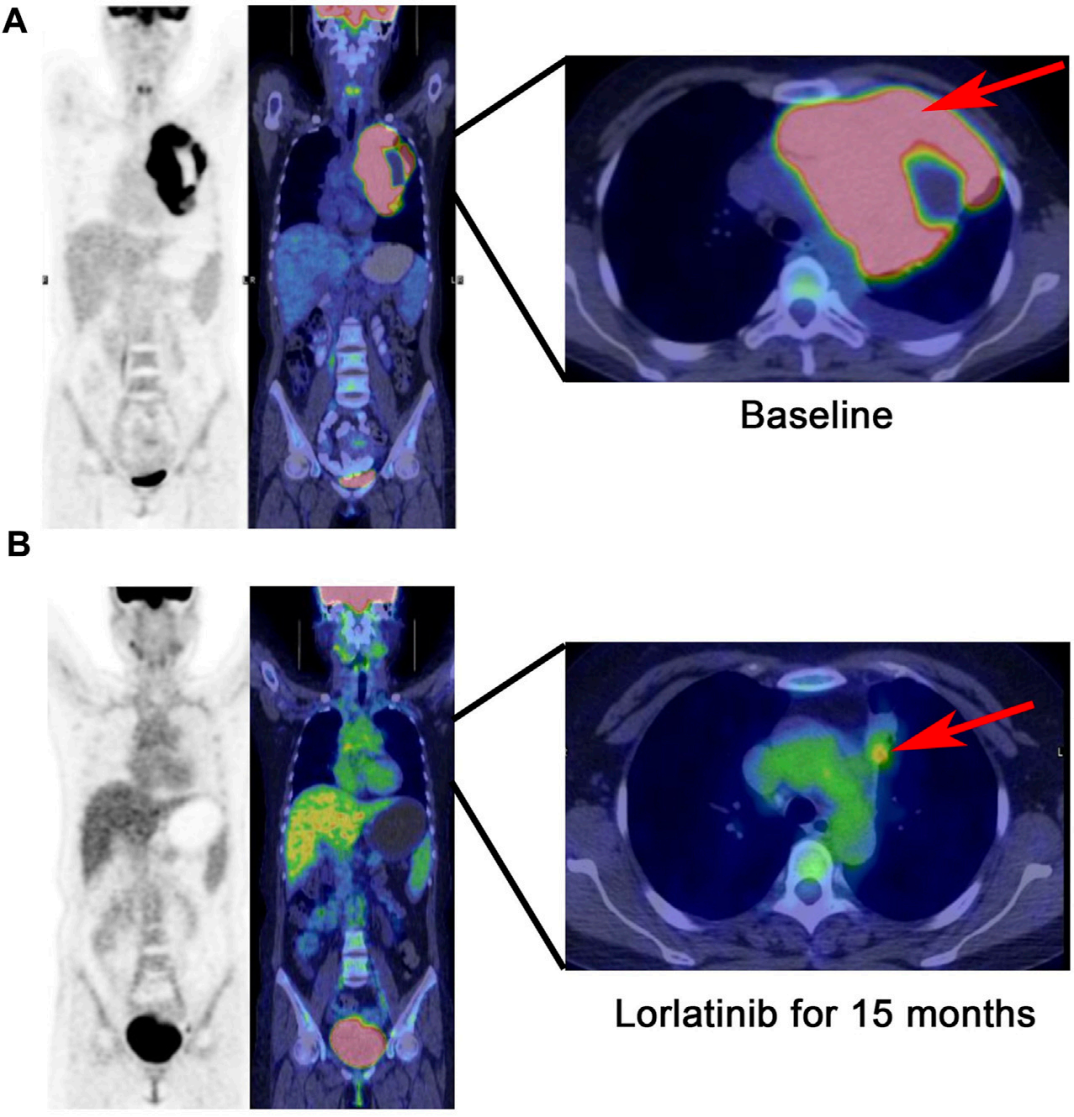
PET-CT scan: The tumor with a size of 14.8 * 9.9 cm is limited to the mediastinum without metastasis at baseline **(A)**, continuous partial response was demonstrated after 15 months following lorlatinib **(B)**.

A 34-year-old female presented to local hospital with productive cough and fever (performance status (PS) score: 3) and was found to have an anterior mediastinal occupation combined pleural effusion on chest computed tomography (CT), measuring approximately 10.1 * 9.4 cm (Figure 1A). Positron emission tomography-computed tomography (PET-CT) scan confirmed the presence of a large irregular mass in the left anterior mediastinum with 14.8 *9.9 cm, showing a high standardized uptake value (SUV) of 29.2 (Figure 2A). In addition, laboratory tests indicated a slightly elevated level of neuron-specific enolase (NSE) at 25.60 μg/L, with the upper limit of the normal range being 16.3 ug/L. The NSE level may be indicative of neurological or neuroendocrine activity and could be relevant to the mediastinal mass. Tissue biopsy suggested an EIMS by immunohistochemistry with cytokeratin (CK) (−), vimentin (Vim) (+), thyroid transcription factor 1 (TTF -1) (+), smooth muscle antibody (SMA) (+). After the confirmation of the pathology, a surgical consultation was sought, and it was determined that the patient was deemed unfit for surgical intervention due to the sizable mediastinal tumor along with a substantial decline in pulmonary function, as indicated by a PS score of 3.

Interestingly, the next-generation sequencing (NGS) conducted on pleural fluid revealed EML4-ALK (E6:A20) fusion with an abundance of 5.26%. Although there was no approved ALK TKI on EIMS, we still considered targeted therapy as first-line treatment. On 24 December 2021, the patient was commenced on lorlatinib (the third generation ALK TKI) 100 mg once daily. After 24 days of lorlatinib treatment, a follow-up chest CT scan revealed that the left anterior mediastinal mass had notably decreased to 6.9 * 5.6 cm (PS score: 1) (Figure 1B). Additionally, the left pleural effusion showed signs of reduction. Following 3 months of lorlatinib treatment, continued tumor regression was evident (Figures 1C, D). A surgical consultation was sought once again. Despite substantial tumor reduction, an improvement in the PS score from three to 1, and notable enhancements in pulmonary function, it was observed that the tumor had infiltrated the major mediastinal vessels, specifically the aortic arch and pulmonary artery, rendering a radical R0 resection unattainable.

**FIGURE 3:**
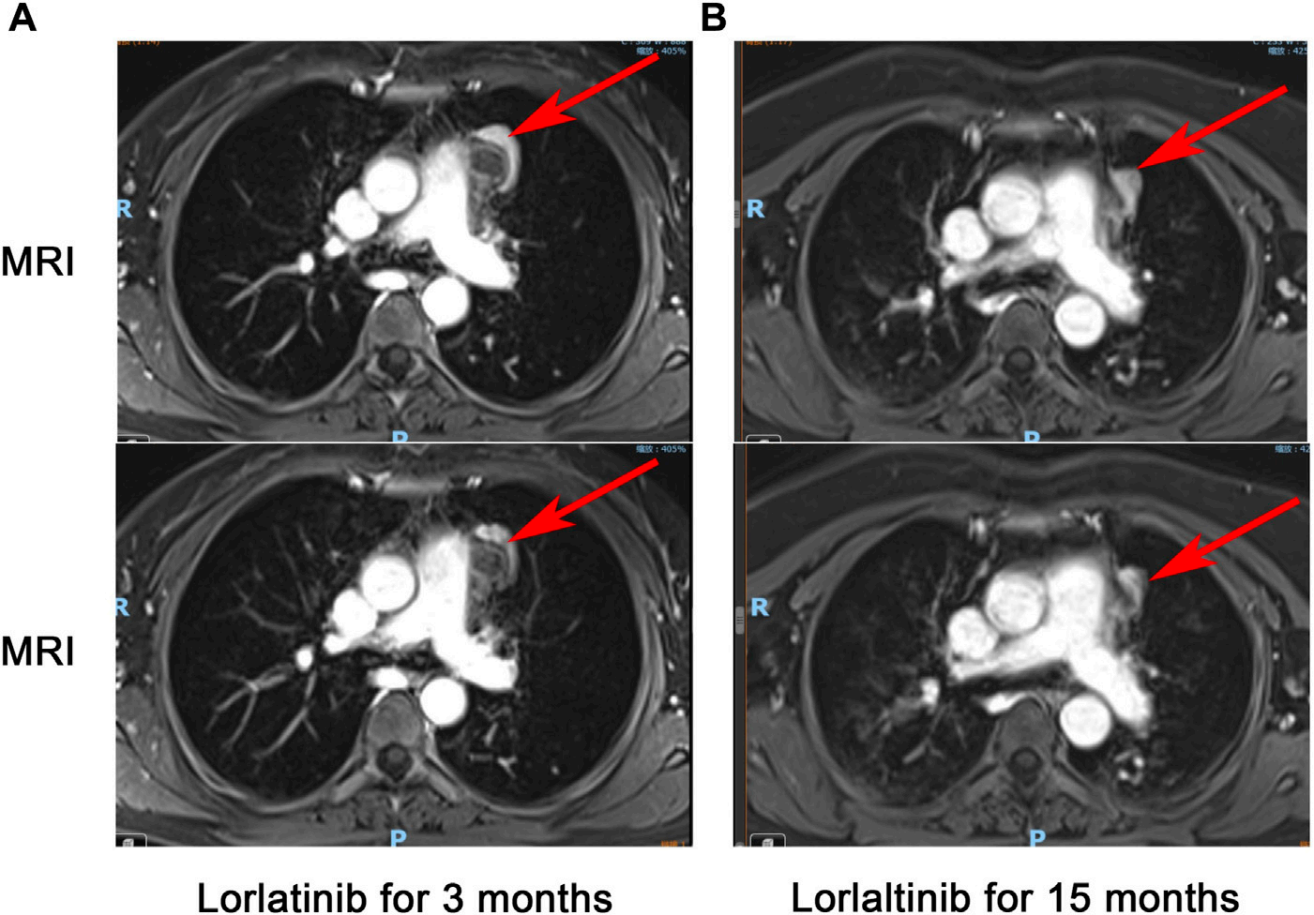
Chest-enhanced MRI with contrast: The tumor infiltrates the mediastinal vessels after 3 months of treatment with Lorlatinib **(A)**, tumor separates from the surrounding blood vessels after 15 months of treatment with Lorlatinib **(B)**.

In March 2023, the follow-up assessment revealed that the tumor size had significantly reduced to approximately 2.3 * 1.3 cm, and SUV value was 4.1 by PET-CT (PS score: 0) (Figure 2B). Chest-enhanced magnetic resonance imaging (MRI) indicated that the tumor was well-defined and separate from the surrounding blood vessels (Figure 3). After consultation with thoracic surgery specialist, the patient underwent EIMS resection on 1 April 2023. Fortunately, postoperative pathological examination revealed fibrosis, necrosis in primary lesions with no tumor cells, consistent with post-treatment changes in inflammatory myofibroblastoma, indicating a pathological complete response (pCR). Also, tissue NGS analysis of the surgical specimen did not detect an ALK fusion mutation. Postoperatively, the patient has continued to receive adjuvant therapy with lorlatinib 100 mg once daily for 8 months without recurrence or metastasis and was continuing with this treatment. Throughout lorlatinib treatment, there was no serious adverse events.

## 3 Discussion

IMT is an infrequent mesenchymal tissue-derived neoplasm, characterized by a low occurrence of lymph node metastasis and distant metastasis. ALK rearrangement is identified in up to 70% or more of cases (Vernemmen et al., 2023). IMT indeed exhibits a preference for certain anatomical sites, including the lung, abdomen, pelvis, and retroperitoneum (Mahajan et al., 2021). EIMS represents a rare subtype of IMT, characterized by a higher prevalence of ALK fusion (Marino-Enriquez et al., 2011). This variant displays a more aggressive and malignant behavior compared to the classic form of IMT. EIMS tends to progress rapidly, with an increased propensity for both recurrence and metastasis. Till now, there is only one documented case in the literature of mediastinal EIMS with ALK fusion reported, where unfortunately, the patient experienced rapid disease progression and succumbed to respiratory failure just 1 day before receiving a formal diagnosis (Pan et al., 2024).

**FIGURE 4:**
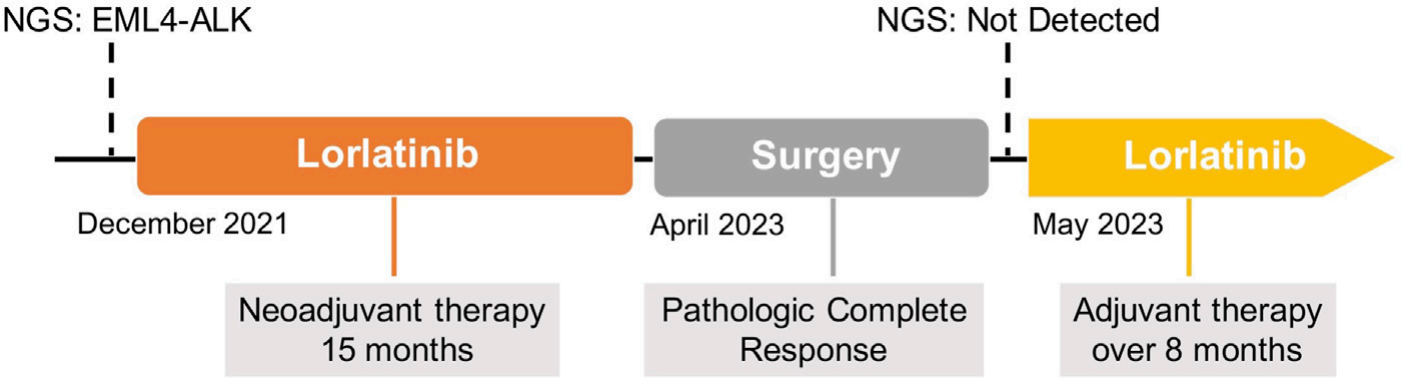
The timeline therapy administration of EIMS with lorlatinib.

In this case, we further validated the effectiveness of ALK TKIs in treating IMT, and EIMS was sensitive to lorlatinib. Furthermore, this case highlights the potential utility of lorlatinib as a neoadjuvant therapy, offering the possibility to convert initially unresectable EIMS cases into resectable ones (Figure 4), thereby substantially enhancing patient prognosis. It may be the first case of lorlatinib in EIMS neoadjuvant therapy.

To our knowledge, several cases of EIMS have been described, and only a few cases show the distant metastasis (Marino-Enriquez et al., 2011; Kimbara et al., 2014; Kozu et al., 2014). Unlike conventional IMT, EIMS is characterized by plump round-to-epithelioid tumor cells embedding in abundant myxoid stroma with inflammatory infiltrate, as well as immunopositivity for ALK, and frequent RANBP2-ALK fusion gene. In 2013, the fourth edition of the World Health Organization (WHO) classification of soft tissue tumors formally recognized EIMS as a variant of IMT. It was identified as having the potential for a more aggressive clinical course, indicating the malignant nature of this tumor (Fu et al., 2015).

The most prevalent fusion observed in EIMS is the RANBP2-ALK fusion (Butrynski et al., 2010). However, in 2017, EML4-ALK fusion was also identified in EIMS (Jiang et al., 2017). As of December 2023, only two cases have documented EML4-ALK fusions, both associated with relatively short overall survival time (Jiang et al., 2017; Pan et al., 2024). As mentioned, ALK TKIs have been extensively used in the treatment of advanced NSCLC. However, their use in the neoadjuvant therapy for NSCLC has been explored in limited retrospective studies (Chaft et al., 2018; Zhang et al., 2019; Zhang et al., 2020). It is worth noting that there is no data or information available regarding the application of ALK-TKI neoadjuvant therapy for mediastinal EIMS. Notably, our case is the first to report a patient with EIMS who achieved a pCR after surgery and has been followed up for more than 8 months post-surgery, demonstrating a survival of more than 2 years.

EIMS typically exhibits a challenging prognosis post-surgical treatment and subsequent chemotherapy and/or radiotherapy. The efficacy of alternative treatment approaches, including radiotherapy, chemotherapy, and steroid therapy, remains uncertain (Sakurai et al., 2004; Fabre et al., 2009). Kurihara-Hosokawa and Fujiya have reported cases of EIMS recurrence where patients have continued to survive with the disease for up to 14 months after undergoing surgical treatment and receiving the ALK inhibitor crizotinib (Kongsgaard et al., 1989; Butrynski et al., 2010). It is worth noting that there are currently three generations of ALK inhibitors in use. Lorlatinib, categorized as a third-generation ALK TKI, was specifically engineered to exhibit comprehensive inhibitory effects on both ALK and ROS proto-oncogene 1, receptor tyrosine kinase (ROS1), while also boasting exceptional capability to penetrate the blood-brain barrier (Basit et al., 2017). Lorlatinib demonstrates significantly greater efficacy than crizotinib in non-small cell lung cancer, with a median progression-free survival (PFS) not yet reached after 5 years of follow-up (Solomon et al., 2024). Given its superior effectiveness in lung cancer, we hypothesize that lorlatinib will exhibit similar efficacy in IMT. Additionally, the utilization of EIMS in conjunction with neoadjuvant therapy was infrequent. There was only one documented case, involving a 17-year-old Japanese boy with locally advanced IMT of the bladder, who received crizotinib as a neoadjuvant therapy, and remained free of recurrence for over a year (Nagumo et al., 2018). This case inspired us to consider the possibility of performing surgery if neoadjuvant lorlaitnib therapy could effectively reduce tumor size. Then we observed that crizotinib as adjuvant therapy was capable of suppressing disease progression in EIMS (Liu et al., 2015). As a result, our patient continued to receive lorlaitnib after surgery without experiencing any recurrence for a period of 8 months. In this case, we have demonstrated the application of lorlatinib as neoadjuvant and adjuvant therapy in mediastinum EIMS, suggesting that the treatment with lorlatinib has continued to be effective in managing EIMS with ALK fusion.

In conclusion, we presented a rare case of pulmonary EIMS. To the best of our knowledge, this may be the first instance of EIMS being treated with lorlatinib as neoadjuvant therapy. Given its rarity, diagnosing EIMS, particularly when it occurs in atypical locations, presents challenges, and should be approached with caution. Pathologists should be aware of EIMS in the respiratory tract and its distinct characteristics to avoid diagnostic pitfalls due to histological similarities with other ALK-positive tumors. It is a case study on lorlatinib specifically, so lorlatinib showed promising results, which opens the possibility of testing other ALK inhibitors as well.

## Data Availability

The original contributions presented in the study are included in the article/supplementary material, further inquiries can be directed to the corresponding author.

## Abbreviations

IMT, inflammatory myofibroblastic tumor; EIMS, epithelioid inflammatory myofibroblastic sarcoma; ALK, anaplastic lymphoma kinase; TKIs, tyrosine kinase inhibitors; EML4, echinoderm microtubule-associated protein-like 4; NSCLC, non-small cell Lung cancer; RANBP2, ran-binding protein 2; RRBP1, ribosome binding protein 1; PS, performance status; CT, computed tomography; PET-CT, positron emission tomography-computed tomography; SUV, standardized uptake value; NSE, neuron-specific enolase; CK, cytokeratin; Vim, vimentin; TTF-1, thyroid transcription factor 1; SMA, smooth muscle antibody; NGS, next-generation sequencing; MRI, magnetic resonance imaging; pCR, pathological complete response; WHO, World Health Organization; ROS1, ros proto-oncogene 1, receptor tyrosine kinase.
